# 3D-Printing Applications in Ostomy Device Creation and Complex Intestinal Fistula Management: A Scoping Review

**DOI:** 10.1055/s-0043-1775748

**Published:** 2023-09-26

**Authors:** Chien Lin Soh, Madhumitha Pandiaraja, Michael P. Powar

**Affiliations:** 1 School of Clinical Medicine, University of Cambridge, Cambridge, United Kingdom; 2 Cambridge Colorectal Unit, Cambridge University Hospitals NHS Trust, Cambridge, United Kingdom

**Keywords:** colorectal surgery, 3D-printing, complex fistula, enterocutaneous fistula, ostomy

## Abstract

**Background** This scoping review aims to provide a summary of the use of three-dimensional (3D) printing in colorectal surgery for the management of complex intestinal fistula and ostomy creation.

**Methods** A systematic database search was conducted of original articles that explored the use of 3D printing in colorectal surgery in EMBASE, MEDLINE, Cochrane database, and Google Scholar, from inception to March 2022. Original articles and case reports that discussed 3D printing in colorectal surgery relating to complex intestinal fistulae and ostomies were identified and analyzed.

**Results** There were 8 articles identified which discussed the use of 3D printing in colorectal surgery, of which 2 discussed ostomy creation, 4 discussed complex fistulae management, and 2 discussed patient models.

**Conclusion** 3D printing has a promising role in terms of management of these conditions and can improve outcomes in terms of recovery, fluid loss, and function with no increase in complications. The use of 3D printing is still in its early stages of development in colorectal surgery. Further research in the form of randomized control trials to improve methodological robustness will reveal its true potential.

The rapid advancements in three-dimensional (3D) 1 printing technology has shown great potential for medicine. 2 From its conception in the early 1980s, 3D printing has evolved into a promising technology with many commercial applications. 3 This method of rapid manufacturing involves the use of software and individual patient data to create a 3D object from a two-dimensional digital image. The process involves acquiring patient information through noninvasive imaging techniques, creation of a 3D model through a computer-aided design (CAD) program, conversion of the CAD file to a printing file (often STL), and printing with a 3D printing technique. 3 There are many types of 3D printing technologies such as vat photopolymerization, material extrusion, and material jetting. 4 5

Patient-specific surgical guides are currently in use in other areas of surgery and in various stages of development. 6 7 Although the applications of 3D printing technology are mostly at the proof-of-concept phase in colorectal surgery, the technology shows promise to transform the practice of management of complex fistulae and ostomy creation. 8 Applications include creation of prosthetics, tools, and reproducing patient-specific anatomy for patient counseling and education and also surgical planning. 9 10 11

3D physical models can be produced based off volumetric scans such as computed tomography (CT), magnetic resonance imaging, and ultrasound data to provide additional information for the surgeon. 12 The use of these models have been widely documented in radiology, with guidance for radiologists published. 13 14 The ability to create personalized and customized items from a wide array of materials is another benefit. The increasing affordability of 3D printing technology also demonstrates its economic feasibility. 4

Although 3D printing research in surgery is mainly focused on plastics, maxillofacial, and orthopaedic surgery, 9 11 15 one sector of medicine seeing great potential benefits is colorectal surgery. Due to the highly variable and complex anatomy of the digestive tract, 3D printing has the potential to assist patients, surgeons, and health care professionals in improving patient outcomes. The area of colorectal surgery has seen the applications of this technology, especially with assisting surgical planning and patient education. 16 Despite discussions by expert groups regarding 3D printing radiological applications in colorectal surgery, specific stoma creation and fistula management was not discussed. 11 Patient-specific anatomical variation can be considered with the design of implants that can be personalized to the complex environment encountered during surgery. 17

Technical challenges of the complex anatomy of the digestive tract and problems faced by patients with stomas and enterocutaneous (ECF) and enteroatmospheric fistulae (EAF) are well documented in the literature. 18 19 However, the application of 3D printing in the area of stoma care and fistula management remains relatively limited, and there is no comprehensive overview regarding stoma creation and complex fistula management and 3D printing in colorectal surgery.

This scoping review encompasses the applications of 3D printing in ostomy creation and ECF management, with its limitations and a brief overview of future recommendations for research.

## Methods

This scoping review was performed in accordance with the Preferred Reporting Items for Systematic reviews and Meta-Analyses extension for Scoping Reviews guidelines.

### Search Strategy

An electronic search of all literature from the earliest available date up to and including March 24, 2022, was done by two independent researchers (M.P. and K.S.). The search terms used were (3D-print* OR additive manufactur* OR 3D print*) AND (“Colorectal Surgery” OR Stoma OR Ileostomy OR Colostomy OR ostomy OR “Rectal cancer surgery” OR Parastomal OR “Enterocutaneous fistula” OR “Enteroatmospheric fistula” OR “Intestinal failure” OR “Colon cancer surgery”). Databases included PubMed, EMBASE, and CENTRAL. A manual search of the references lists of articles found through the original search was done to identify any further relevant studies.

### Selection of Relevant Studies

All primary research articles reporting the use of 3D printing in colorectal surgery or colorectal surgical patients were included. Studies were excluded if: (1) they were performed in an animal model; (2) no full-text article was available; (3) the full-text article was in a language other than English; (4) they were a review article; and (5) they described applications of 3D printing for surgical education and training.

Following removal of duplicate abstracts, the remaining titles and abstracts were screened independently by two reviewers (C.S. and M.P.). Any disagreements between the two reviewers were resolved by automatic inclusion. Potentially eligible studies were then retrieved for full-text assessment. All full texts of retrieved articles were read and reviewed by two authors (C.S. and M.P.) to determine studies meeting the eligibility criteria. Where there was discrepancy, a third reviewer (M.P.P.) made the final decision.

### Data Charting

Characteristics of the included studies were summarized by two authors (C.S. and M.P.) in a Microsoft Excel data extraction spreadsheet designed a priori and modified iteratively. The information extracted included: the primary author, study design, year of publication, country of study, study population size, name of 3D printer, printer name, type of surgical procedure concerned, type of 3D printing utilized, 3D printing method description, name of software used for product design, printing material used, reported outcomes, cost, main challenges reported, and future areas of study proposed. The descriptive abstracted summaries of the included articles were reviewed and the two authors (C.S. and M.P.) met to generate the core themes, which were: (1) applications of 3D printing in stoma management, (2) applications of 3D printing in ECF management, and (3) applications of 3D printing in patient education relating to stoma or ECF management.

## Results

**Fig. 1:**
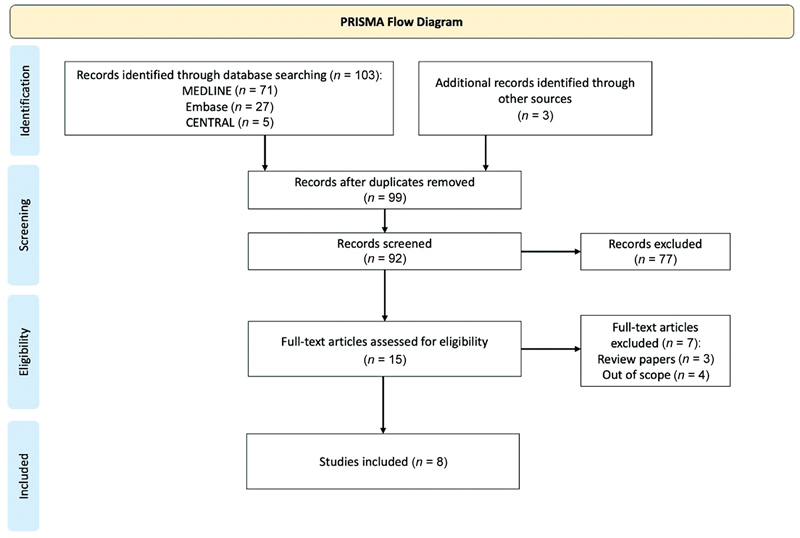
Preferred Reporting Items for Systematic reviews and Meta-Analyses (PRISMA) flowchart. A flowchart describing the screening process of the articles, with *n* = 8.

The literature search identified 106 articles, of which 92 were screened following removal of duplicates and exclusion of non-English articles. In total, 8 articles met the inclusion and exclusion criteria. Fig. 1 illustrates the study selection process.

Characteristics of the included studies are summarized in Table 1 . Most of the included studies were conducted in Asia (Japan, Taiwan, and China, *n* = 5), 20 21 22 23 24 with the others completed in Spain ( *n* = 2) 25 26 and the United States ( *n* = 1). 27 Four studies were of descriptive case study design, 22 23 24 27 two were quasi-experimental clinical trials, 25 26 and two were laboratory research studies. 20 21 Sample sizes were small across the reviewed literature, with only three studies reporting the use of 3D-printed device in more than one patient. 23 25 26

**Table 1:** Characteristics of articles included in the scoping review

Author	Year	Country	Type of study	Sample size	Disease of interest
Cosman et al ^27^	2021	USA	Case report	1	Colostomy
Tominaga et al ^23^	2016	Japan	Case report	5	Ileostomy and colostomy
Zahia et al ^26^	2022	Spain	Quasi-experimental	9	Colostomy, ileostomy, or urostomy
Yeh et al ^20^	2021	Taiwan	Laboratory research	0	Colostomy
Xu et al ^22^	2019	China	Case report	1	Enteroatmospheric fistula
Durán Muñoz-Cruzado et al ^25^	2020	Spain	Quasi-experimental	4	Enteroatmospheric fistula
Huang et al ^24^	2017	China	Case report	1	Enterocutaneous fistula
Hu et al ^21^	2021	China	Laboratory research	0	Enteroatmospheric fistula

Note: Articles and their details included within the review with basic details.

The applications of 3D printing described in the literature can be broadly divided into three categories: production of paraphernalia to aid self-management of stoma, 23 27 production of ostomy appliances, 20 26 and production of appliances for the management of EAF or ECF. 21 22 24 25 The key findings of the included articles are detailed in Table 2 .

**Table 2:** Key findings of included studies

Study	Application of 3D printing	Software applications used	Type of 3D printing	3D printer utilized	Material used	Main findings
Cosman et al ^27^ (2021)	Fabrication of flange stabilizer to help with ostomy patch adhesives changes in individuals with impaired dexterity to minimize risk of stoma leakage	Fusion 360	Material extrusion	Prusa i3 MK2S	Polylactic acid (PLA)	General findings: • Ostomy aid would be especially beneficial to ostomates with impaired dexterity • Model published on open-access site that anyone with access to a 3D printer can use to print this device Advantages: • Low cost of production (< $1) Limitations: • PLA neither autoclavable nor dishwasher safe • Device optimized only for specific flange size • Durability of device
Tominaga et al ^23^ (2016)	Creating stoma and face plate models for patient education to improve daily stomal care and reduce complications such as stoma-associated skin problems	Geomagic Free Form graphics	Vat photopolymerization	Objet 260 Connex	—	General findings: • 3D-printed models useful for patient education on stomal care, especially so for patients may find it challenging to learn new skills • Models facilitate discussions at staff conferences and help in finding solutions to stoma-related issues • Could lead to less skin problems associated with daily stomal care Advantages: • Self-reliance in stomal care • 3D models can be kept for use at home or in future outpatient clinic visits Limitations: • High cost (∼$100USD per patient) • Lengthy process • Change in stoma size during perioperative period
Zahia et al ^26^ (2022)	Production of customized ostomy pouch adhesives to reduce the occurrence of complications associated with change in parameters of ostomy over time and enhance patient quality of life	3D Slicer, Creo Parametric, Meshlab	Vat photopolymerization	—	—	General findings: • Feasible to use low-cost manual scanners to create 3D-printed stoma barrier rings with little difference in quality and reliability Advantages: • Several benefits of using handheld scanner over CT scan for design of personalized patch: reduced need for hospital resources and appointment, less complex data files, quicker processing in design software Limitations: • Poor adhesion of ostomy pouch adhesives to skin • Reported patch rupture and skin irritation • Concerns regarding patch thickness compared with commercial patches
Yeh et al ^20^ (2021)	Production of customized colostomy base plate to minimize stoma leakage	Not specified	Material extrusion	—	Thermoplastic elastomer (TPE)	General findings: • It is possible to calibrate 3D printing parameters to achieve softness using low-cost materials Advantages: • Manufacturing methods are modifiable to achieve desirable properties in 3D-printed devices Limitations: • Only manufacturing materials, methods and 3D printing parameters discussed in this study
Xu et al ^22^ (2019)	Fabrication of fistula stent for EAF plugging to accelerate fistula closure	SolidWorks	Material extrusion	—	Thermoplastic polyurethane (TPU)	General findings: • Novel 3D-printed fistula stent can significantly minimize volume of enteric effluent loss and speed up rehabilitation Advantages: • Enteric effluent loss markedly reduced after stent placement • Stent acts as temporary tract, allowing restoration of enteral nutrition Limitations: • Changes in the morphology and size of fistulous tract during therapy • Stent may enlarge EAF and reduce likelihood of spontaneous closure • Limited sample size
Durán Muñoz-Cruzado et al ^25^ (2020)	Manufacturing customized prostheses for patients with complex EAF	FreeCad 0.16, Meshmixer, Regemat 3D designer software	Material extrusion	Regemat 3D	Polycaprolactone (PCL)	General findings: • Feasible to design, produce, and use a customized device that suits the patient's wound through 3D printing to achieve a “floating stoma” Advantages: • Reduced enteric effluent and associated skin complications • Increased patient comfort and reduced need for analgesia • Device generation process took 4 ± 0.45 h Limitations: • Issues with adherence between PCL material and negative pressure wound therapy system that the stoma was connected to
Huang et al ^24^ (2017)	Fabrication of patient-personalized fistula stent for ECF	SolidWorks	Material extrusion	—	Thermoplastic polyurethane (TPU)	General findings: • Feasible to produce a stent specific to patient's fistula anatomy to reduce loss of intestinal effluvium, patient nutrition and recovery, and local wound inflammation Advantages: • Less mechanical damage to surrounding mucosa compared with other plugging methods • Reduction in loss of enteric effluent and greater tolerance to physical rehabilitation • No endoscopic-guided implantation of stent required Limitations: • Limited sample size
Hu et al ^21^ (2021)	Preparation of personalized stents for intestinal fistulas	Creo Parametric, CloudCompare	5 + 1-axis 3D printing	—	Thermoplastic polyurethane (TPU)	General findings: • 5 + 1-axis 3D printing method devised through the study showed greater efficiency, created better stent surface properties and shape, and optimal mechanical strength and elasticity for human bioprostheses Advantages: • Better microstructure of stent produced with five-axis printing method, reducing likelihood of intestinal fluid leak and electrolyte loss • Orderly channel arrangement in stents allowing uniform adhesion of cells Limitations: • Potential utility, safety and efficacy of stents in vivo yet to be proven • Biocompatibility of fistula stent with intestinal wall fibroblast lines yet to be studied

Abbreviations: 3D, three-dimensional; CT, computed tomography; EAF, enteroatmospheric fistulas; ECF, enterocutaneous fistulas.

Note: Describing studies and the main findings from each paper.

### Applications of 3D Printing in Patient Education of Stoma Management

There were two studies identified describing the creation of product to aid patient education and daily stomal care. Cosman et al presented the design and production of a flange stabilizer that would help patients with impaired dexterity in performing daily ostomy bag changes correctly, thus reducing the chance of leakage due to improper pouch reattachment after emptying. 27 In a similar vein, Tominaga et al described the use of 3D-printed stoma models and baseplates to allow patients to practice cutting their ostomy baseplate with the 3D stoma model to minimize skin irritation associated with suboptimal daily stomal care. Stoma care was found to be improved in all 5 patients trained using the 3D-printed models. 23

### Applications of 3D Printing in Production of Ostomy Appliances

Two studies described the use of 3D printing in producing customized constituents of the ostomy system. 20 26 Zahia et al conducted a clinical trial of the 3D-printed ostomy skin barrier to evaluate the performance and safety of the devices. The study reported the feasibility of using low-cost scanners over computed axial tomography (CT scans) to obtain 3D images of the abdominal surface to design and print ostomy pouch adhesives without compromising the reliability of the patch. Issues reported by the patients included poor adhesion of the patch, patch rupture, skin irritation, and inadequate thickness, but no differences were observed in the incidence of these between the patches printed using different scanning modalities. 26 Yeh et al described an experiment using the Taguchi method to optimize 3D printing parameters that would allow low-cost materials to achieve the appropriate softness needed for an ostomy bag. 20

### Applications of 3D Printing in Production of Fistula Stents

Four studies explored the applications of 3D printing in producing stents for the management of EAF or ECF. 21 22 24 25 All of the studies describe EAF and ECF as challenging conditions with high associated morbidity if not managed properly initially. Given the immense variability in the morphology of the fistulous orifice in patients, the necessity of a tailored approach has been highlighted. 22 24 25 The 3D-printed fistula stent was reported to be effective in controlling intestinal effluvium and achieving wound isolation in conjunction with negative pressure wound therapy. 25 Hu et al described the development of a novel 5 + 1 axis 3D printing platform to achieve more high-precision, customized intestinal fistula stents. 21 Scanning electron microscope analysis of stents produced with the traditional three-axis printing method and those produced with the five-axis printing method showed superiority of the latter stents in terms of regularity of the stent surface. The stents printed using the new five-axis printing method devised were reported to be less likely to lead to intestinal fluid leakage and electrolyte loss following implantation, as well as provide a more favorable surface for adhesion of cells from the intestinal wall. 21

### 3D Printing Technology

In terms of the 3D printing technology employed, material extrusion, also known as fused deposition modeling ( *n* = 5) and vat photopolymerization ( *n* = 2) were most commonly used. The material used for device production was highly variable, as different material properties were needed for the device described in each study. Among the four studies that described the fabrication of a stent for EAF or ECF management, three of them used thermoplastic polyurethane (TPU). This was attributed to the biocompatibility and flexibility of the material. 21 22 24 Several factors are important to consider during material selection for medical device production using 3D printing, including the physical properties of the material, its printing difficulty, cost, sustainability, and environmental impact of the chosen material. 20 The adherent property of the material used was also noted to be poor by several authors and suggested the need for further developments in this area. 25 26

### Challenges and Recommendations

**Fig. 2:**
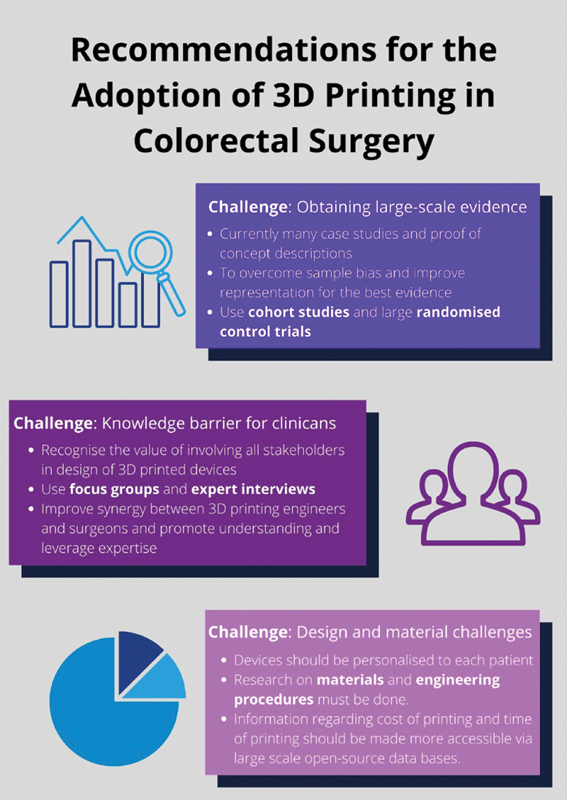
Graphic on challenges and recommendations. The figure describes the challenges and solutions for implementation of three-dimensional (3D) printing in colorectal surgery.

Fig. 2 summarizes the main challenges and respective recommendations developed from the literature with regards to future research being conducted in the field of 3D printing and their applications in colorectal surgery.

### Quality of Studies Found

Most research was low-grade with seven of the publications at or below level III evidence. No randomized control trials (RCTs) were found with full information. A Cochrane-registered trial was identified investigating the effect of having a preoperative education session with the fitting of a 3D-printed stoma on the patients' quality of life compared with felt-tip marking of the ostomy site. However, this study was excluded as it is still ongoing with no results reported yet. 28

## Discussion

This scoping review provides the first summary of the applications of 3D printing in the field of device creation in ostomy and ECT and EAF fistula management within the field of colorectal surgery. The current level of knowledge remains limited with only a small number of studies identified, despite there being a larger base of literature for other applications of 3D printing within colorectal surgery with two existing systematic reviews. Although the review's results demonstrate successes within different approaches in the field, these must be considered in the context of their limitations such as small sample sizes and recent applications. Many applications in colorectal surgery remain at the “proof-of-concept” stage, and literature remains limited to case reports. More focused research and larger scale trials involving larger patient groups must be conducted to progress to adoption of these applications in clinical practice. Health care professionals must be prepared to adopt 3D printing into health practice, as usability and cost-effectiveness of these technologies increase with new developments in the field, and to shape the new landscape in which 3D printing is used in medicine. Current abdominal appropriateness criteria and guidelines by specialist groups have not addressed our applications beyond anal fistulae. 1

The studies could be subdivided into ostomy, ECF/EAF fistula, and patient education devices. Most devices were designed to be personalized to each patient and used TPU as materials. The specific technologies used were diverse, with a wide range of techniques and approaches employed to design each device. However, the principles of 3D printing remained consistent throughout, with many different modalities used to derive information via scanning, design of the device, and subsequent printing and application.

### Ostomy

Zahia et al show that ostomy pouch adhesives can be created via 3D printing technology from information derived from different 3D scanning modalities. 26 Yeh et al demonstrate the design of a low-cost ostomy bag using 3D printing, although it was not tested on any patients. 20 These studies demonstrate the proof-of-concept ideas that can be applied to future studies with patient cohorts.

### Fistula

The management of complex ECFs had the highest number of case reports, with a majority originating from Asia. The reports describe the use of material extrusion alongside thermoplastic urethane and polylactic acid to custom design patches that were personalized to each patient's fistula. These proof-of-concept studies seek to block the EAF to manage the condition in the short and long term, although challenges included wide clinical variability and the device potentially changing the shape of the wound. 21 22 24 25

### Patient Models

Cosman et al describe a 3D-printed flange stabilizer for patients with spinal cord injury. 27 Tominaga et al describe a case in which 3D-printed stoma models are used to discuss care and problems with patients. 23 This study highlighted the effectiveness of 3D-printed models in teaching patients with dementia management of ostomy devices. 3D-printed models can facilitate self-management of ostomies in patients who might otherwise struggle due to their physical or mental disabilities, which can lead to better compliance, patient autonomy, and outcomes.

## Advantages and Disadvantages

3D printing has many advantages to traditional methods of device creation in the context of colorectal surgery. Advantages described in the included papers were reduced hospital length of stay, quicker recovery, and restoration of bowel function through the use of an ostomy. Patients were able to be discharged at an earlier date and this has additional advantages for the hospital. The studies also identified the quick process of creation and manufacturing the devices using 3D printing, alongside the availability of the materials used. Most studies described using thermoplastic materials, which has mechanical strength, flexibility, and biocompatibility. 29 One study described modifications in printing technique to optimize the material properties of the devices used to fabricate the products. The general desirable characteristics were materials that were lightweight and strong to allow patients to continue with normal life with comfort and reduce the strain placed on the delicate tissues. Much consideration is given to the setup of the 3D printers and the thickness and infill settings, alongside optimization of the software files used. The files were stored digitally and modified as required, with changing patient measurements. This customization allows a more accurate fidelity and representation of the device required for the patients. The accuracy of the replication of these models are yet to be studied, with potential error factors such as limitations of the technology and model inaccuracies. 8 In addition, cost considerations and waste reduction using the process of 3D printing in comparison to traditional manufacturing methods makes it an advantageous avenue to continue research. Cost savings in other fields of medicine such as orthopaedic surgery and general medicine have been attributed to reduction in procedural and operative times, although one must be careful with extending this assumption to colorectal surgery as the needs for printing applications vary. 30 31

Nevertheless, the limitations of 3D printing technology are that it is yet to be employed on a wider scale in clinical practice. A common drawback observed in the articles included issues with fit within changing anatomy and compromised viability of the skin and soft tissues. The relative inaccessibility of the software used to novices or untrained clinicians, as noted in many of the studies involved collaboration with engineering departments and expert consultants, may be a potential barrier to its adoption. The process of designing a customized product with complex dimensions without the necessary engineering expertise may be a long and arduous process. Although open-source designs exist for free online, concerns regarding lack of regulation and expertise limit its use. Studies on the viability and long-term safety of these devices are yet to be conducted.

## Publication Bias

A recognized source of bias in reviews is publication bias, which describes the trend that studies with statistically and clinically significant findings tend to be more likely to be published than their counterpart with no or little significant findings. In addition, the potential detrimental statistical false positive influence of publication bias, particularly in small to medium samples, together with high heterogeneity in study methods and 3D printing methods, has meant that conducting a meta-analysis would have not been feasible.

## Future Directions

The lack of literature and robust evidence available on 3D printing in colorectal surgery is a significant limitation. A common issue is the lack of patient data and the low level of evidence, as many of the studies were case descriptions with single individuals. It is not possible to extrapolate such data, and more trials and studies must be conducted with 3D printing and colorectal surgery. A recommendation for future research is to automate the 3D printing process for the novice user, and to make these technologies available in developing countries as an accessible low-cost tool. 25 26

## Conclusion

This scoping review has reaffirmed that 3D printing has a promising role in terms of management of complex intestinal fistulae and in ostomy creation. It has been shown to improve outcomes in terms of recovery, fluid loss, and function with no increase in complications. However, the use of 3D printing in colorectal surgery remains nascent and further research in the form of RCTs to improve methodological robustness will reveal its true potential.
